# Cellular and Molecular Mechanisms of Environmental Pollutants on Hematopoiesis

**DOI:** 10.3390/ijms21196996

**Published:** 2020-09-23

**Authors:** Pablo Scharf, Milena Fronza Broering, Gustavo Henrique Oliveira da Rocha, Sandra Helena Poliselli Farsky

**Affiliations:** Department of Clinical and Toxicological Analyses, Faculty of Pharmaceutical Sciences, University of São Paulo, São Paulo, SP 005508-000, Brazil; scharfpablo@gmail.com (P.S.); milenafbroering@gmail.com (M.F.B.); gustavohorocha@gmail.com (G.H.O.d.R.)

**Keywords:** environmental pollutants, xenobiotics, hematopoiesis, myelotoxicity

## Abstract

Hematopoiesis is a complex and intricate process that aims to replenish blood components in a constant fashion. It is orchestrated mostly by hematopoietic progenitor cells (hematopoietic stem cells (HSCs)) that are capable of self-renewal and differentiation. These cells can originate other cell subtypes that are responsible for maintaining vital functions, mediate innate and adaptive immune responses, provide tissues with oxygen, and control coagulation. Hematopoiesis in adults takes place in the bone marrow, which is endowed with an extensive vasculature conferring an intense flow of cells. A myriad of cell subtypes can be found in the bone marrow at different levels of activation, being also under constant action of an extensive amount of diverse chemical mediators and enzymatic systems. Bone marrow platelets, mature erythrocytes and leukocytes are delivered into the bloodstream readily available to meet body demands. Leukocytes circulate and reach different tissues, returning or not returning to the bloodstream. Senescent leukocytes, specially granulocytes, return to the bone marrow to be phagocytized by macrophages, restarting granulopoiesis. The constant high production and delivery of cells into the bloodstream, alongside the fact that blood cells can also circulate between tissues, makes the hematopoietic system a prime target for toxic agents to act upon, making the understanding of the bone marrow microenvironment vital for both toxicological sciences and risk assessment. Environmental and occupational pollutants, therapeutic molecules, drugs of abuse, and even nutritional status can directly affect progenitor cells at their differentiation and maturation stages, altering behavior and function of blood compounds and resulting in impaired immune responses, anemias, leukemias, and blood coagulation disturbances. This review aims to describe the most recently investigated molecular and cellular toxicity mechanisms of current major environmental pollutants on hematopoiesis in the bone marrow.

## 1. Hematopoiesis Overview

### 1.1. Hematopoiesis and Hematopoietic Hierarchy

Hematopoiesis is a continuous, albeit complex, process that aims to generate blood cell subtypes in a steady manner. Hematopoietic stem cells (HSCs) represent a small population of pluripotent, self-renewing cells responsible for initiating the renewal of blood cells by giving rise to other cell progenitors. In humans, such cells are CD34^+^CD38^−^ [1].

In bone marrow (BM), HSCs initially give rise to multipotent progenitors (MPPs), which can also be considered pluripotent. These cells have limited self-renewal capabilities, yet possess full-lineage differentiation potential [2]. These cells remain mostly quiescent at the G0 phase of the cell cycle [2,3], but through signaling mediated by intrinsic and extrinsic factors, this population initiates cell cycle entry and starts differentiating [4,5]. MPPs give rise to common myeloid precursors (CMPs) and lymphoid precursors (CLPs) through cytokine signaling and the activation of several transcription factors [6]. MPPs differentiated into CLPs originate lymphocytes and natural killer cells that rely mainly on activation of PU.1, Ikaros and GATA-3 transcription factors [7]. Soluble factors such as IL-7 and its receptor (CD127) actively participate in CLP maturation and development, as IL-7 and CD127 deficiencies disrupt production of B and T cells. On the other hand, MPP fate-decision differentiation into CMP, which originates granulocyte-macrophage (GMP) and megakaryocyte-erythrocyte progenitors (MEPs), is modulated by PU.1 and GATA-1 [7,8,9].

GMP differentiation is dependent on secretion of granulocyte-macrophage-colony-stimulating factor (GM-CSF), after which macrophage-colony-stimulating factor (M-CSF) modulates the differentiation of monocytes/macrophages and granulocyte-colony-stimulating factor (G-CSF) modulates the differentiation of neutrophils, basophils, and eosinophils; the latter in a process known as granulopoiesis. MEP, under erythropoietin modulation (EPO), initiates erythropoiesis originating erythrocytes and, under thrombopoietin (TPO) effects, MEP originates megakaryocytes and platelets [6,10]. During erythropoiesis, MEP differentiates into burst-forming unit erythroid (BFU-E) and, finally, into colony-forming unit erythroid (CFU-E); this whole process is tightly modulated by soluble mediators such as erythropoietin (EPO), stem cell factor (SCF), and IL-3 and -6. At a molecular level, activation of GATA-1, STAT-5, and Kruppel-like factor-1 (KLF-1) pathways ensures that erythroid differentiation and maturation take place. Disruption of these molecular pathways leads to anemia and myeloproliferative syndromes [6,11,12].

### 1.2. HSC Quiescence

The functionality of HSCs depend on the balance between quiescence and activation. Reduced ability of HSCs to leave quiescence results in insufficient blood cell production; on the other hand, greater amounts of HSCs leaving quiescence or failing to return to quiescence after activation exhaust the HSC pool, leading to BM failure, which favors the onset of malignant diseases [13,14]. Proper response by hematopoietic progenitors to regulatory stimuli and adequate control of cell-signaling pathways that culminate in controlling DNA damage are essential for avoiding exhaustion of the HSC pool [15].

Quiescent HSCs eventually become senescent and lose the ability to proliferate. The fine-tuning between proliferative, quiescent, and senescent cells is vital for the homeostasis of the hematopoietic environment [16]. In specific areas of BM, approximately 80% of HSCs remain quiescent throughout an average human lifespan, ensuring their “stemness” when needed. HSCs can leave quiescent states and become proliferative in a transient manner in response to external stimuli, such as injuries or infections, then become quiescent again [17]. The modulation of proliferation, differentiation, and migration capabilities of HSCs is essential for control of their quiescence. The hematopoietic microenvironment is fundamental for such regulation, fine-tuning the balance required for overall homeostasis [13].

### 1.3. HSC Niches

HSCs are spatially distributed in BM in highly organized niches composed of several cell subpopulations that maintain HSC quiescence. Interactions with several stromal adjacent cell populations such as fibroblasts, osteoblasts, macrophages, and endothelial cells, in addition to the actions of soluble factors released by these cell groups, ensure the preservation of the HSC pool and modulate proliferation and quiescence of these cells [18].

A significant amount of HSCs is also associated with sinusoidal endothelial cells, from where they are able to readily enter the peripheral blood [19,20]. The release of these cells into the bloodstream and their subsequent migration and return to BM are physiological processes pivotal for homeostasis [21,22].

HSCs are located both in the endosteal and vascular niches. The endosteal niche is a complex structure that includes several components, such as progenitor and stromal cells, growth factors, and extracellular matrix molecules that participate in the regulation of hematopoiesis [23]. The endosteal niche is interposed between the bone and BM, and includes bone-forming osteoblasts and bone resorption osteoclasts alongside other cells, such as fibroblasts, macrophages, and endothelial cells located near the endosteum [23,24]. The anatomical location of the endosteal niche also supports the modulation of HSCs, as the endosteum provides a microenvironment containing low levels of oxygen, which is an important factor for HSC quiescence [25]. Osteoblastic cells present in the endosteal niche secrete chemical mediators that activate cell signaling cascades and regulate HSCs [26,27]. The secretion of TPO and angiopoietin (Ang-1) leads to the expression of adhesion molecules in HSCs (β1-integrin and *N*-cadherin) and increases quiescence of these cells [26,27]. Osteoblasts also secrete Notch receptor ligands and soluble Jagged factors; the activation of Notch receptors in HSCs inhibits differentiation of these cells and increases their self-renewal capacity under either stress or physiological conditions [27,28].

In the vascular niche, hematopoiesis occurs in the extravascular spaces between the sinuses. The medullary vascular sinuses are lined with endothelial cells and are surrounded by adventitious cells, also called CXCL12 abundant reticular (CAR) cells [29]. CAR cells are the major producers of cytokines that modulate HSC behavior, such as CXCL12 and SCF [29]. The proximity between sinusoidal endothelial cells and HSCs is very important for the maturation of the latter and, therefore, for the hematopoietic process [30]. The vascular niche is essential for the production of factors essential for the mobilization, homing, and engraftment of HSCs. The interaction between mature cells and the vascular niche is required for the release of these cells into the bloodstream. The expression of adhesion molecules by endothelial cells, such as vascular cell-adhesion molecule-1 (VCAM-1), associated with chemokine factors, mediates the maturation of megakaryocytes and the release of platelets into the bloodstream [31].

BM shelters several populations of progenitor cells other than HSCs that are essential for homeostasis, such as mesenchymal stem cells (MSCs). These cells provide a supportive microenvironment for HSCs and display high regenerative capabilities with a therapeutic potential, arising from several cell lines such as adipocytes, chondrocytes, and osteocytes [32]. The therapeutic effects associated with paracrine mechanisms linked to MSCs are extremely complex and include diverse cytokines and growth factors as well as other related receptors and signaling molecules with a wide range of biological functions [33]. MSCs and their derivatives are also essential for maintaining hematopoiesis, and for the maturation of hematopoietic lineages [34,35].

Endothelial progenitor cells (EPCs) can also be found in BM; EPCs and HSCs share hemangioblasts as common progenitors [36]. EPCs are recruited in response to ischemia and initiate angiogenesis, leading to the formation of new blood vessels, connecting fibronectin, and forming colonies and/or colony-forming units [37,38]. EPCs resemble embryonic angioblasts, which are anchorage-independent cells capable of proliferating, migrating, and differentiating into mature ECs [39]. EPCs express mainly cluster of differentiation 34 (CD34) and fetal liver kinase 1 (FLK1), although other markers have also already been identified, such as vascular endothelial growth factor receptor 2 (VEGFR2) and CD146 [40]. EPC mobilization into the bloodstream occurs depending on different disease conditions, such as tumor and cardiovascular disorders, and is mediated by a plethora of chemical mediators, with VEGF being the most well-known [41].

**Figure 1:**
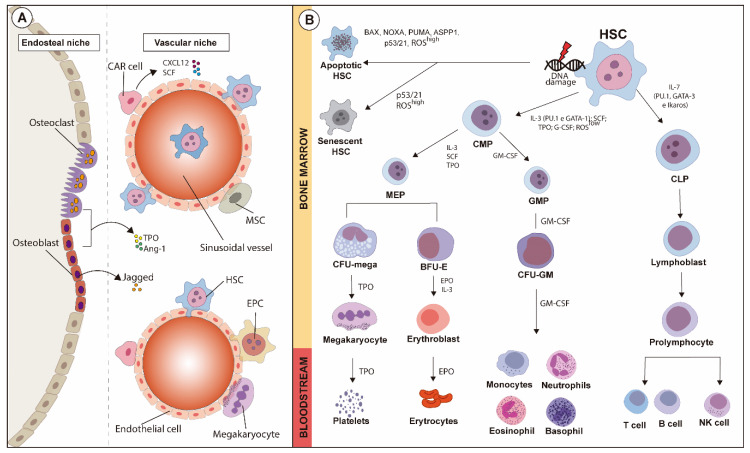
Schematic overview of HSC niches and fate-decision of hematopoietic lineages during hematopoiesis, along with the major transcription factors and cytokines involved. (**A**) HSC niches comprise endosteal and vascular regions containing several cell types. In the endosteal niche, osteoclasts and osteoblasts, as well as other cell types, support the vascular niche where mesenchymal stromal cells (MSCs), hematopoietic stem cells (HSCs) and CXCL12-abudant (CAR) cells are anchored. The maturation of hematopoietic lines is modulated by the fine balance between both niches. (**B**) Hematopoietic stem cells (HSCs) can undergo either apoptosis or senescence after suffering oxidative stress or DNA damage, which might or might not activate apoptotic pathways. Under physiological status, activation of GATA-1 and PU.1, low levels of reactive oxygen species (ROSs), and modulation exerted by soluble factors such as IL-3, SCF, TPO and G-CSF in HSCs give rise to common myeloid progenitors (CMPs), which then originate neutrophils, eosinophils, basophils and monocytes. On the other hand, common lymphoid progenitors (CLPs), under influence of IL-7, are modulated by the activation of GATA-2, Ikaros, and PU.1. CMPs originate erythrocytes and platelets after the maturation of intermediary precursors, and CLPs mature into B and T lymphocytes and natural killer cells.

A schematic overview of HSC niches is illustrated in Figure 1A.

### 1.4. HSC Mobilization

The recruitment of hematopoietic stem cells and progenitors (HSPCs) from BM into peripheral blood is a process known as mobilization that occurs either under physiological or stressful conditions, such as acute inflammation and chemotherapy [42,43]. Mobilization occurs based on the interplay between HSPCs and BM niche components, as well as resulting from the interaction between chemokines and their receptors. Adhesion molecules, proteases, and activation of intracellular signaling pathways also play roles in cell mobilization [42].

Indeed, the inactivation of stromal cell-derived factor-1 (SDF-1) and interleukin-8 (IL-8) alongside the enzymatic actions of proteases such as elastase, cathepsin G, and metalloproteinases (MMP-2 and MMP-9) modulate the SDF-1/C-X-C receptor 4 (CXCR4) axis and promote the release of BM-resident progenitor cells to peripheral tissues [44,45]. The SDF-1/CXCR4 axis is crucial for the maintenance, retention, and mobilization of HSPCs under homeostatic conditions or after an injury [46], as HSPCs that express CXCR4 are attracted to the highly expressing SDF-1 endosteal niche. Also, upregulation of adhesion molecules such as vascular cell adhesion molecule-1 (VCAM-1), very late antigen 4 (VLA-4), and leukocyte function antigen 1 (LFA-1) are important for the retention of HSCPs in BM; in turn, downregulation of these molecules and of other chemokines is closely related to the mobilization of progenitors into the bloodstream [42].

HSPCs are endowed with a great therapeutic potential and, as with most of BM transplants, rely on the use of HSPCs isolated from peripheral blood after the mobilization of progenitors has been induced. The modulation of the aforementioned mechanisms mobilizes HSPCs, and this practice is widely used in clinical practice [47]. G-CSF is one of the most effective mobilizing agents [47], and repetitive G-CSF stimulation accelerates myeloid hematopoiesis, enhancing HSPC motility and migration into peripheral areas of the body [45]. Also, G-CSF induces the release of proteases, cathepsin G, and MMP-9, which downregulate levels of adhesion molecules, favoring the mobilization of HSPCs [48].

Increased release of progenitor cells and mature leukocytes from BM is part of the immune response leading to inflammatory processes. Depending on the severity of the inflammatory response, immature cells are also delivered into the bloodstream [49]. A broad range of molecules such as leukotrienes, resolvins, annexin A1, cytokines, and chemokines, among others, are involved in modulating inflammation and returning HSPCs to BM [50,51,52,53]. After an injury, such as exposure to radiation, stromal cells in BM release high amounts of SDF-1, which is responsible for inducing homing and modulating the repopulation capabilities of HSPCs [54].

### 1.5. DNA Damage, ROS Generation and Hypoxia in the Control of HSC

The mechanisms underlying the transformation of normal cells into malignant phenotypes are not yet fully understood. In order to better understand such mechanisms, the cell cycle of hematopoietic cells has been extensively investigated, as aging processes are closely linked to the accumulation of DNA damage and telomerase shortening in quiescent cells [4,55]. Such accumulated DNA damage in quiescent HSCs leads to the activation of DNA repair mechanisms only when these cells leave the G0 phase of the cell cycle; failures of DNA repair machineries, however, allow such accumulation of genomic damage to deregulate further cell cycle processes, and this is a key mechanism for leukemogenesis [15,56,57]. Still, in order to avoid malignant changes and to sustain homeostasis, hematopoietic progenitors are capable of tolerating some DNA damage due to protective mechanisms, such as DNA translesion synthesis (TLS) [58,59].

The accumulation of DNA damage and the insufficient activation of repair mechanisms leads to the exhaustion of progenitors, driving them to senescence or apoptosis. Activation of apoptotic pathways in HSCs aims to avoid a proliferation of compromised cells that have suffered irreparable DNA damage, preventing the development of malignancies. The main pro-apoptotic factor activated is the tumor suppressor protein p53. Activation of p53 leads to quiescence via activation of its downstream effector and cell cycle inhibitor p21, causing damaged cells to cease proliferation and become senescent [60,61]. Induction of apoptosis is linked to pathways involving BAX, NOXA, and PUMA in addition to activation of the ASPP1 protein, a p53 cofactor that selectively promotes or inhibits p53-mediated apoptosis. The process of controlling apoptosis and activation of HSCs, while complex, is crucial for preserving the HSC pool [61,62,63].

DNA damage also activates other protective intracellular mechanisms, such as autophagy. Autophagy is a catabolic process marked by the lysosomal degradation of damaged organelles and proteins, and is essential for maintaining hematopoiesis and HSC differentiation and quiescence by suppressing cell metabolism [64,65,66]. In response to stressors, HSCs induce autophagy to prevent cell death, mitigate the increase of reactive oxygen species (ROS) levels and promote activation of DNA repair machineries [67,68]. Mice lacking the autophagy related gene 7 (atg7) are more susceptible to oxidative stress, accumulation of DNA damage and loss of function of HSCs after irradiation [69].

Much like apoptosis, generation of ROSs also plays a protective role and induces autophagy. However, oxidative stress, when excessive, becomes one of the main causes of DNA damage. Excessive production of ROSs and the resulting imbalance of enzymatic mechanisms that constitute redox signaling leads to oxidative stress, resulting in oxidative-induced damage [70]. Uncontrolled ROS production is responsible for damaging DNA and macromolecules such as lipids and proteins [71,72]. Several studies associate high levels of ROS and oxidative damage as factors for the initiation and progression of hematological diseases, such as leukemias [73,74,75,76]. Still, ROS generation is essential for the functionality and modulation of HSC phenotypes and their progenitors, as the generation of ROSs regulates the self-renewal and differentiation of adult stem cells [77]. At low levels, ROSs ensure that precursors remain pluripotent, but at excessive levels, ROSs can impair the functionality of these populations, even leading to BM failure under extreme circumstances [78,79].

Aiming to avoid excessive oxidative stress, antioxidant proteins act quickly in the face of high levels of ROSs. Among them, superoxide dismutase (SOD), catalase, and glutathione peroxidase are the most important; reduced glutathione (GSH), which also exists in the cell in its oxidized form (GSSG), is perhaps the most abundant of enzymatic antioxidants [80,81].

The enzymatic machinery responsible for keeping ROS levels low in hematopoietic progenitor cells is also aided by the microenvironment of hematopoietic niches [25,78]. As the generation of ROSs is closely linked to oxygen demand, several transcription factors are involved with the regulation of low levels of oxygen in HSCs. Hypoxia-inducible factor 1 (HIF-1) is the main transcription factor responsible for allowing cells to adapt to low oxygen levels; the expression of its inducible subunit, HIF-1α, is linked to the maintenance of ROSs at homeostatic levels. Lacking activity of HIF-1α in progenitor cells leads to the exacerbated generation of ROS, culminating in increased cell proliferation and loss of self-renewal capacity [82,83]. Activation of fork-head O transcription factor (FoxO) subunits also plays a crucial role in keeping low the levels of ROSs in HSCs, thus playing a protective role upon oxidative stress. The deletion of heterodimers of the FoxO’s family in hematopoietic cells substantially decreases the number of HSCs, leading to increased cell cycle activation, and this effect appears to be dependent on oxidative stress, as treatment with n-acetylcysteine, a potent antioxidant, is able to restore homeostatic cell-cycling conditions [84,85]. Activation of p38 mitogen-activated protein kinase (MAPK), a transcription factor, in response to ROS generation, is also important for regulating HSCs in the face of oxidative stress. MAPK activation limits the lifespan of progenitor cells, and inhibition of p38 pathways protects the self-renewal capabilities of HSCs, avoiding their exhaustion and thus being a potential therapeutic target for improving the “stemness” of HSCs [79,86].

These reports highlight the complexity of the processes responsible for regulating HSC physiology in hematopoiesis and how fragile this vital system is. Moreover, the maturation and differentiation of hematopoietic precursor cells leading to the delivery of mature cells into the bloodstream also involves fine-tuning mechanisms, which can be easily disrupted. Disturbances on HSC biology and functions and on the delivery of precursors into the bloodstream are mechanisms of blood and vascular diseases. The hematopoietic hierarchy alongside indications of HSC damage leading to senescence and apoptosis is summarized in Figure 1B.

## 2. Cellular and Molecular Mechanisms of Toxicity on Hematopoiesis

The amount of pollutants in the environment has increased in the modern era. Air pollution before industrialization mainly originated from the burning of organic materials used for house-heating and cooking. Such pollution resulted in increased releases of carbon monoxide and particulate matter, which by then already affected the health of populations [87]. Since industrialization, the amount and diversity of pollutants in the air have increased, and alarming levels of pollutants have been quantified in several countries worldwide, regardless of them being developing or having already been developed [88,89]. Consequently, hazardous effects for both the environment and human health have led to public health concerns worldwide. The complexity of hematopoiesis and of the fine-tuning mechanisms that control functions of blood components such as host defense, oxygen supply for tissues, and bloodstream rheology makes the hematopoietic system a suitable target for the actions of xenobiotics, including those released to the environment. Blood is also a pivotal matrix used for the assessment of xenobiotic levels and biological end points during intoxications. Below is described the most relevant data on the cellular and molecular actions of current environmental pollutants on the BM hematopoietic system in mammals.

### 2.1. Benzene and Its Metabolites

Benzene (BZ) is a volatile liquid aromatic hydrocarbon solvent, a byproduct of petroleum refinement. It used to be widely employed as an industrial chemical, either as a solvent or as a starting material for the synthesis of other chemicals, until its severe toxicity to occupationally exposed humans, especially by inducing hematological disorders and cancer, was described [90,91]. While the relationship between BZ exposure and leukemia had already been reported at the beginning of the 20th century in industry workers [91], BZ was classified as an environmental carcinogen in 1982 due to its BM toxicity [92]. Nowadays, even though the use of BZ in working environments is controlled around the world [93], alarming levels of this solvent are frequently found in the air of both developing and developed countries [94,95]. BZ, alongside toluene and xylene, is a contaminant of gasoline and diesel, thus being a common pollutant in high-traffic cities [96,97]. Smokers are also exposed to high amounts of BZ and its metabolite hydroquinone (HQ), which are compounds of cigarettes [98,99].

BZ is extensively metabolized after absorption via lungs, mouth, and skin, and the resulting metabolites are responsible for its harmful effects. BZ is initially converted to benzene oxide by cytochrome P450 enzymes, mainly CYP2E1, in the lungs and liver; benzene oxide is then rearranged into phenol, which is subsequently metabolized to HQ, catechol, and 1,4-benzoquinone (1,4-BQ) by action of CYP2E1 enzymes, especially in the liver. HQ is transported to the BM and metabolized to the highly reactive BQ by oxidative enzymes, such as myeloperoxidases (MPOs) [100]. Benzene oxide can also be hydrolyzed generating catechol and 1,2-BQ, or it can be metabolized by glutathione S-transferases forming the less-toxic metabolite *S*-phenylmercapturic acid. Benzene oxide can also have its aromatic ring opened, resulting in reactive muconaldehydes and *E*,*E*-muconic acid [100], which are quantified in urine as intoxication end-points. Detoxification by redox systems in BM, such as NADPH–quinone oxidoreductase 1 (NQO1), reduces the local levels of oxidative toxic agents generated by BZ metabolism [100,101], but prolonged exposure to BZ leads to lasting high levels of BZ and accumulation of BQ, which lead to toxic effects in BM. The role of redox systems on BZ toxicity has been evidenced by several reports as lower and higher hematotoxicity caused by BZ in CYP450 and NQO1 knockout mice, respectively [102,103]. NQO1 polymorphism is associated with increased hematotoxicity in humans exposed to BZ [104], and administration of the major compounds of garlic, diallyl di and trisulfide reduces BZ hematotoxicity in mice by deactivating CYP2E1 and MPO and activating GSH and NQO1 [105].

BZ exposure affects BM by interfering with different hematopoiesis pathways due to actions of its multiple metabolites, mainly HQ and BQ. This leads to failures in the BM environment, which result in decreased peripheral counts of erythrocytes, leukocytes, platelets, pancytopenia, aplastic anemia, myelodysplasia, and myelogenous leukemia [90,100,106,107]. Robust studies carried out over the years have evidenced that BZ exposure affects BM cells by causing chromosomal aberrations, gene mutations, oxidative stress, apoptosis, epigenetic deregulation, impairment of DNA repair, modification of protein secretion, and suppression of immune systems [90,91]. It is worth mentioning that toxic effects are not due to direct actions of BZ and its metabolites only, as these are also powerful inducers of ROS generation, and high ROS levels also mediate the harmful effects of BZ intoxication [90].

It is worrisome that there are no described safe levels for BZ exposure regarding hematopoiesis. BZ toxicity is influenced by different genetic profiles and living conditions; blood cell functions and their production and delivery processes are very intricate and offer a great number of targets for BZ metabolites to act upon [101,108]. Novel toxic mechanisms exerted upon hematological parameters by BZ exposure have been continuously reported, especially for exposures at levels considered sub-toxic [108,109].

As the overall population should not be exposed to high levels of BZ, elucidating how BZ exposures of low frequency and low doses disturb cell pathways is a goal of current investigations. Indeed, gene alterations in human HSCs have been fully demonstrated after low-dose exposures to BZ (lesser than 1 ppm, the threshold level), resulting in aberrant expression of downstream genes, malignant transformations, and HSC dysfunction [110,111,112]. Exposure of HSCs to non-cytotoxic doses of BZ have caused ruptures of DNA structures at breakpoint hot stops in the leukemia-related genes MLL and CBFB similar to those found in leukemia patients [113]. DNA breaking within the MLL gene leads to rearrangement of over 120 other partner genes, resulting in acute leukemia with poor prognostics [114], and DNA breakings within the CBFB gene is the cause for about 10% of acute myeloid leukemia cases [115]. Enhanced autophagy is another mechanism elicited by lower levels of BZ exposure, as reported in BM mononuclear cells from patients exposed to BZ and in mice cells exposed to HQ. Autophagy is regulated by post-translational modifications such as phosphorylation, ubiquitination, and acetylation. BZ exposure reportedly decreases acetylation of autophagy components by inhibiting the activity of acetyltransferases such as p300 [116]. Hematopoietic cells collected from workers exposed to low doses of BZ or incubated with 1,4 BZ have shows increased apoptosis and autophagy rates, dependent on expression of the long-non-coding RNA cRNAVNN3 triggered by BZ or 1,4 BZ-induced oxidative stress; cRNAVNN3-enhanced phosphorylation of Bcl-2 and beclin-1 led to cell death [117]. Physiological autophagy levels are required for maintaining stable cell homeostasis under stress conditions, while exacerbated autophagy induces uncontrolled cell death. Therefore, depending on the intensity of the aggressive stimuli, autophagy can be either beneficial or harmful, playing either a protective role on cell death or contributing to it [118].

HIF-1α is a transcription factor involved in the harmful effects of BZ on HSC niches. HIF-1α controls the hypoxic microenvironment of such niches as to maintain quiescence, survival, and metabolic phenotypes of cells by increasing anaerobic glycolysis and reducing ROS generation [78,82,119]. Recent evidence has demonstrated that exposure to BZ can inhibit HIF-1α, as mice exposed to BZ showed high levels of ROSs alongside lower levels of HIF-1α in niches of stem cells [120]. HIF-1α binds to DNA in the hypoxia-response-element (HRE) DNA domain, and several genes such as those responsible for the expression of vascular endothelial growth factor (VEGF) and erythropoietin (among others) contain HRE binding sites in their sequences and are thus targets for the actions of HIF-1α. It has been demonstrated that treatment of BM cells from mice with BZ downregulated expression of genes containing the HRE domain, impairing expression of genes involved with self-renewal, cell cycle, differentiation, and apoptosis pathways of HSCs [121]. In accordance, overexpression of HIF-1α in a myelogenous leukemia (CML) K562 cell line reduced apoptosis and ROS levels induced by 1,4-BQ by targeting Nox4, Bcl-2, and key glycolytic enzymes [122].

Reduction of the number of circulating red blood cells is also a clinical symptom of low intensity BZ toxicity [123]. It has been demonstrated that damage caused to erythroid burst-forming units (BFU-E) in workers exposed to BZ is dose-dependent, and that erythroid cell differentiation is more sensitive to the harmful effects of BZ or HQ exposure than other hematopoietic precursor cells [124,125]. It has also recently been reported that BZ inhibited erythroid cell differentiation by downregulating the expression of miRNA-451a and miRNA486-5p [126]; miRNA-451a positively modulates the terminal differentiation of erythroid cells protecting red cells against oxidative stress [127,128] and miRNA486-5p regulates the differentiation and growth of erythroid cells [129].

Another topic of BZ toxicity that is yet to be fully elucidated is whether hematopoiesis disturbances caused by BZ exposures of low frequency and dose are reverted after exposure ceases. It has been demonstrated that exposure to BZ in mice impaired frequency and colony formation of HSC SCA-1^+^c-kit^+^ cells (LSK) and reduced mRNA levels of Notch-1 and p53 in BM cells collected 10 months after the end of BZ exposure. This provides evidence that molecular and cellular alterations affecting the self-renewal of HSCs are long-lasting after exposure to BZ, and may allow pre-leukemic clones to evade elimination, leading to an increased risk of development of transforming neoplasia [130]. In the same vein, exposure of rats to BZ at low doses for 14 days impaired erythropoiesis, which partially recovered 56 days after exposure has ceased as reduced numbers of reticulocytes in the bloodstream and reduced phagocytosis ability in macrophages collected from mature erythroblastic islands could be observed after this period [131].

The modulation of the cytosolic transcription factor aryl hydrocarbon receptor (AhR) linked to BZ toxicity on BM and blood cells has recently been investigated. AhR is a ligand-activated transcription factor expressed in hematopoietic progenitor cells, lymphocytes, neutrophils, and splenocytes [132,133,134]. The connection between BZ exposure and AhR was first demonstrated by studies that provided evidence that hematopoietic toxicity induced by BZ was not observed in AhR knockout mice, but that toxicity would take place when the BM of animals was repopulated with cells from wild-type mice. Accordingly, BZ-induced hematotoxicity in irradiated wild-type mice repopulated with AhR knockout BM cells was not observed, as measured by granulo-macrophage-colony-forming units assay [135,136,137].

AhR expression is also essential for the differentiation and activation of Th17 cells in the pathogenesis of rheumatoid arthritis [138,139]. Solid experimental and clinical data show a correlation between exposure to cigarette smoking and the induction and aggravation of rheumatoid arthritis [140,141]; there is also increased AhR expression in the synovial membrane of smokers [142]. Cigarette smoke is an important source of BZ and HQ, as each stick delivers about 72.2 and 100 μg of BZ and HQ, respectively [99,143]. The connection between HQ exposure and AhR to rheumatoid arthritis was recently tested in mice and rats exposed to HQ by inhalation following an experimental design of low-dose exposures. Although HQ-exposed animals had no alterations in BM or blood cell numbers, disease symptoms worsened, with a high frequency of AhR^+^ neutrophils and Th17 lymphocytes in the inflamed synovia. Accordingly, rheumatoid arthritis symptoms were not observed in AhR knockout mice exposed to HQ [133,134] (Heluany et al., under review). These data evidence that exposure to BZ metabolites worsens rheumatoid arthritis involving HQ actions through AhR on blood cells, and that both BZ and HQ are cigarette compounds involved with the harmful effects on the evolution of the disease as a result of cigarette smoking.

Although scientific studies regarding hematotoxicity evoked by BZ exposure have been published extensively in scientific literature over the last 50 years, the broad actions of BZ and its metabolites upon the complex hematopoietic phenomena and resulting effects to the immune system in exposed subjects, especially those exposed to low concentrations, constitute a still-vast area for investigations with a meaningful impact on public health.

### 2.2. Engineered Nanoparticles

Nanotechnology as a field has grown at a fast pace over the last 20 years, and has been responsible for the development of materials with useful properties for a plethora of industrial and biological applications in the engineering, communication, food, and textile industries, to name a few. Nanomaterials also have bioenvironmental applications, and can act as reliable drug carriers [144]. Nanoparticles (NPs) are sized less than 100 nm, and are characterized by an increased surface area and unique physicochemical properties, which make them extensively employed in several fields. Due to NPs being such a recent breakthrough, the number of studies focusing on subjects occupationally exposed to NPs is still low, but increasing. The impact of NPs on occupational health and safety is currently difficult to predict, halting advances on the risk assessment of NPs [145]. The main factors that determine the toxicological effects of NPs are exposure conditions such as route, concentration, and duration; the individual characteristics of exposed subjects; and the intrinsic characteristics of NPs, including ability to bind to or coat surface species, surface area, composition, and catalytic activity, among others [146]. Moreover, NP pharmacokinetics differ from those of common bulk materials, which is another barrier to advances on understanding their toxicological potential [147].

Current nanomaterial research has mostly focused on nanotechnology applications, whereas there is little information regarding occupational and environmental exposure assessment and risk characterization associated with NPs. As airborne NPs are mainly absorbed by respiratory routes, some of the toxic effects of NPs on lungs have been described in both humans and experimental animals, being characterized mostly by inducible inflammatory reactions [144]. Still, inhaled NPs have a very small size and can be arrested from alveoli into other tissues by the bloodstream, which may lead to systemic harmful effects [148]. Indeed, it has been demonstrated that pulmonary instillation of titanium dioxide (TiO2) NPs has resulted in increased plaque deposits in atherosclerosis-prone apolipoprotein E-deficient mice [149]. In addition, environmental exposure to TiO2 NPs has affected the biology of circulating angiogenic cells (EPCs), which contribute to the harmful vascular effects of NPs [150]. After birth, EPCs circulate in the peripheral blood until being recruited and incorporated into sites of active neovascularization, being then committed to vascular repair [37,151].

No clear evidence is available regarding the effects of occupational or environmental exposure to NPs on hematopoiesis in BM. Nevertheless, alterations in the number and functions of circulating immune cells have been described in addition to those induced by the inflammatory response by itself as a result of exposure to NPs. Magnetic NPs have been extensively used as contrasting agents in magnetic resonance imaging; assessment of toxic effects due to the inhalation of manufactured magnetic NPs has revealed these NPs to be highly distributed to different tissues and to increase hematopoiesis in the spleen, where there are increased numbers of erythroid and myeloid cells in the red pulp [152].

Although studies available on the harmful effects of engineered nanotechnology products on the hematopoietic system are scarce, the systemic toxic effects of NPs that unintentionally reach complex tissues (such as the central nervous system [153]) have shown evidence that further investigations on the toxic potential of NPs on BM could provide valuable data for risk assessment studies.

### 2.3. Incidental Environmental Nanoparticles and Particulate Matter

Exposure to NPs or particulate matter (PM) found in polluted air causes systemic harmful effects, which have been extensively described in humans, and exposure to associations of NPs and PM can lead to the onset of diseases as well [154,155,156]. PM comprises a heterogeneous mixture of particles of different sizes and chemical compositions. The severity of toxic systemic effects is closely linked to particle size, as both NPs and PM 2.5 (smaller than 2.5 μm) can reach the bloodstream from alveoli and be distributed into tissues causing systemic effects [157,158,159,160]. Particles can also contain harmful airborne microorganisms and metals, which greatly increase their toxicity [161,162]. The World Health Organization estimates 92% of the global population lives in areas where the levels of fine PM 2.5 exceed the recommended annual average air concentration limit of 10 μg/m^3^. Recent findings have shown that exposure to PM 2.5 can pose hazards to public health at low levels, even below those recommended by regulatory agencies [154,163,164,165,166,167].

Local and systemic inflammation, especially linked to chronic exposures, is a common hallmark of exposure to airborne NPs and MPs [160,168]. Cytokines secreted at inflammation sites reach BM and alter HSC niches, affecting maturation and differentiation phases of leukocytes and delivery of cells into the bloodstream. Both acute and chronic in vivo exposure to PM are reported to cause lung inflammation and leukocytosis due to an increased release of immature granulocytes into circulation [169]. This effect was evidenced by increased numbers of band neutrophils found in the bloodstream and a reduced monocyte transit time in BM after lung instillation of PM 10 in rabbits; this effect was also observed in rabbits instilled with supernatant culture medium of alveolar macrophages incubated with PM 10, but not in rabbits instilled with supernatant collected from alveolar macrophages incubated with inert carbon [169,170]. Thus, PM 10 actions depend on activation of lung macrophages, followed by secreted chemical mediators reaching BM and inducing neutrophil and monocyte delivery into the bloodstream [171,172].

The association between PM 2.5 exposure and cardiovascular morbidity and mortality has been extensively demonstrated in epidemiological and experimental studies [173,174]. Both direct and indirect actions of these particles on BM have been shown. Toxic effects on the cardiovascular system caused by MP 2.5 are characterized by alterations on blood rheology and endothelial cells of blood vessels, mostly associated with endothelial oxidative stress and inflammation [175,176]. Moreover, it a tracheal instillation of PM 2.5 from diesel exhaust in mice was reported to have increased the mobilization of neutrophils from BM, and the resulting neutrophilia led to myocardial oxidative stress [177]. Solid evidence has demonstrated that in vivo exposure to PM 2.5 affects BM by limiting the inherent functionality and mobilization of EPCs into the bloodstream, as acute exposure of humans or mice to PM 2.5 has been negatively correlated with plasma levels of EPCs [178,179]. In addition, EPCs from the BM of mice exposed to PM 2.5 have displayed reduced proliferation and tube formation capabilities that were not due to cell death, but rather dependent on the downregulation of genes that support cell proliferation and the cell cycle of BM EPCs. These effects were absent in overexpressing extracellular superoxide dismutase ecSOD-Tg mice exposed to PM 2.5, resulting in reduced oxidative stress and higher nitric oxide bioavailability even upon exposure to PM 2.5. GSH levels were also reduced in BM cells obtained from PM 2.5 exposed mice [179]. Therefore, harmful effects on BM EPCs caused by exposure to PM 2.5 may comprise likely mechanisms for its toxic effects linked to cardiovascular morbidity and mortality.

Other studies further evidenced the toxic effects of PM on BM cells, as incubation of PM 2.5 or PM 10 collected from polluted air impaired proliferation of human HSCs in a concentration-dependent manner, increased expression of cytokine genes associated with inflammation (e.g., TNF-α and IL-6), and impaired expression of the cell cycle regulator gene p53 [180].

Recent studies have described associations between exposure to PM 2.5 during pregnancy and effects on HSCs in the BM of offspring. Pups from female mice exposed to PM 2.5 via respiration during pregnancy suffered from lung inflammation and oxidative stress, which lasted until adulthood. Although offspring aged two and six months had normal leucograms, the two-months-old offspring showed increased BM oxidative stress, inflammation, and osteoclast activity. Damage done to BM evolved and six-months-old offspring exhibited senescent phenotypes of BM HSCs, demonstrated by reduced clonogenic formation, donor-cell-derived reconstitution and self-renewal, increased levels of mitochondrial ROSs, NrF2 expression, cyclin-dependent kinase inhibitors, and increased p38 phosphorylation and DNA double-strand breaking. Considering that no effects were observed on the BM of exposed dams, it is likely that offspring from mothers exposed to PM 2.5 have less efficient anti-oxidant mechanisms in their BM than their mothers [181].

Although scientific studies assessing exposure to PM and BM effects are not plentiful, this only reifies that the BM microenvironment should be considered for risk assessment studies. BM is a direct target for the actions of PM, especially PM 2.5, enduring impairments on cell proliferation, lifespan, and functionality due to systemic oxidative stress and inflammation.

### 2.4. Dioxins and Polychlorinated Biphenyls (PCBs)

Dioxins are persistent organic pollutants (POPs) released to the atmosphere as undesired byproducts of an anthropogenic and natural origin. These compounds can originate as byproducts from combustion processes, such as the incineration of solid waste, the chlorine bleaching of paper and wood-pulp, the burning of coal in power plants, and forest wildfires [182]. These pollutants also occur as contaminants in several pesticides, herbicides, and fungicides [183]. Among POPs, dioxins are considered the most hazardous to human health, with their major toxic effects being linked to binding to the aryl hydrocarbon receptor (AhR) in several cell types.

PCBs make up a class of 209 volatile, structurally related organochlorine compounds used for different industrial and commercial applications, which were mass-produced until the 1970s. Since then, PCBs have been identified as carcinogens, and their global commercial production was banned by signatory nations in the Stockholm Convention on Persistent Organic Pollutants in 2001 [184,185]. Nevertheless, PCBs continue to pose a significant risk to human health through exposure sources such as the continuous release from hazardous waste sites, PCB-contaminated equipment that is still in use, and contact with construction materials used in buildings erected prior to the PCB production ban. In addition, non-legacy PCB congeners have also been detected in paint and industrial pigments [186]. PCBs are still considered among the most important groups of food contaminants, and food and agricultural authorities strictly monitor the PCB contamination of foods. As air pollutants and contaminants of extensively used organochlorine pesticides, PCBs contaminate foods in the same manner as described above, by being byproducts of industrial processes, the combustion of certain materials, or accidental fires. Recycling and the production of certain minerals can also produce PCBs [187]. Depending on the position of chlorine atoms in the biphenyl backbone, PCBs can be of planar or non-planar geometry. Non-chlorine substitution in the ortho position determines non co-planar geometry, while substitution of one to four chlorine molecules in the ortho position determines varying degrees of co-planar ring geometry. Co-planar congeners bind to AhR and are therefore called dioxin-like PCBs. Among 209 known PCB congeners, 12 can bind to AhR [188]. Long degradation half-life and high liposolubility contribute to the bioaccumulation of dioxin and PCBs in biological organisms [189].

AhR is a cytoplasmic basic helix-loop-helix/PAS transcription factor that, upon activation by ligands, can lead to either physiological or toxic effects. AhR has traditionally been described as a central regulator of responses to environmental factors and of xenobiotic metabolism. First studies on the toxic effects of dioxins and AhR evidenced 2,3,7,8-tetrachlorodibenzo-p-dioxin (TCDD, dioxin) can bind to AhR, leading to several toxic effects [190,191]. Under homeostatic conditions, AhR remains predominantly in the cytoplasm as part of a protein complex linked to molecular chaperone heat shock protein 90 (HSP90), p23, and XAP2 [192]. Several stressors can drive AhR activation and evoke its conformational transition, resulting in its nuclear translocation [193]. AhR then dissociates from HSP90 and binds to the AhR nuclear translocator (ARNT), and the AhR/ARNT complex binds to promoter regions in the DNA known as AhR-responsive DNA elements or xenobiotic response elements (XREs), which leads to an increased expression of target genes (e.g., cytochrome P450 (CYP) 1A1, CYP1A2 and CYP1B1) [193,194]. This canonical pathway for AhR activation mediates several toxic responses, including liver damage, chloracne, teratogenesis, cancer, and immunosuppression [195,196].

In recent years, research has demonstrated that AhR-null mice suffer from developmental impairments, evidencing AhR as a crucial homeostasis modulator in several tissues and biological processes, including hematopoiesis [197,198]. AhR modulation on HSC self-renewal, proliferation, cell cycle, and senescence has been demonstrated by the in vivo gene deletion of the AhR exon 3. HSCs from AhR knockout mice abandon quiescence and become hyperproliferative, revealing that AhR is likely a negative regulator of excessive or unnecessary proliferation [197,198,199]. AhR also affects the maturation of HSCs by acting on progenitor strains. AhR deletion in human embryonic stem cells, or blocking of these cells with AhR antagonists, leads to increased differentiation into CD34^+^, CD45^+^, and CD31^+^ EPCs [200,201]. Conversely, in later maturation phases, AhR deletion favors the proliferation of myeloid colonies [201].

Epidemiologic studies have demonstrated associations between 2,3,7,8-tetrachlorodibenzo-p-dioxin (TCDD), the most toxic member of the polychlorinated dibenzodioxins (PCDD) family, and onco-hematologic diseases, particularly non-Hodgkin lymphomas, chronic lymphocytic leukemia, and multiple myeloma [202,203,204]. Indeed, the BM of adult mice exposed to acute doses of TCDD becomes hypocellular, with a significant decrease in the total number of HSCs—effects that are not due to cell death, but rather to direct AhR modulation [205].

Exposure to TCDD impairs humoral responses, and it has been determined that such exposure can lead to B-cell disorders and increased incidence of B-cell-derived cancers [206,207]. Mice exposed to TCDD have shown a reduced number of B-cell progenitors [208] and skewed HSC differentiation favoring myeloid progenitors to the detriment of lymphoid progenitors, giving rise to mature B cells [110,209]. It has also been reported that HSCs treated with TCDD are not able to repopulate the BM of irradiated mice [210,211]. Gene analysis of HSCs revealed that TCDD treatment modified the transcription of genes linked to both migration—such as CD184 (CXCR4) and CD44—and the development and function of the hematological system (e.g., Fos, JunB, Egr1, Ptgs2, and CXCL2) [212].

TCDD is not only toxic to HSC, and its effects on BM stromal cells can also contribute to decreased B-cell numbers. Exposure to TCDD downregulates IL-6 gene transcription in stromal cells, inhibiting the growth of early B-cell progenitors in a NF-kB-dependent manner [213]. Inefficient humoral responses in offspring from mothers exposed to TCDD and dioxin-like PCBs has led to the reprogramming of hematopoietic stem and progenitor cells during development [214].

The toxic effects of dioxins upon the hematological system demonstrate the importance of AhR on the control of proliferation, function, and migration of hematological progenitor cells in BM. Even though dioxin-like xenobiotics are capable of strongly binding to AhR, and of causing severe toxic effects in humans, the harmful effects caused to the hematological system, mainly impairing humoral responses, are suitable end points for the risk assessment of dioxin exposure.

### 2.5. Heavy Metals

The number of individuals exposed to has metals increased throughout history in accordance with industrial and urban growth, eventually leading to researchers investigating correlations between exposure to metals and the onset of diseases [215]. Heavy metals (HMs) are raw materials of great importance for the steel cutting, welding, electroplating, plastics, and automobile industries [216]. HMs are classified by their atomic number, atomic weight, density, and toxicity, of which chromium (Cr), lead (Pb), mercury (Hg), cadmium (Cd), arsenic (As), copper (Cu), manganese (Mn), nickel (Ni), zinc (Zn), and silver (Ag) are the most relevant for human exposure and toxicity [217]. Among these HMs, Pb, Cd, As, and Hg have been described as harmful to BM, causing anemia and immune deficiencies, as evidenced by epidemiological and clinical studies and by experimental models of intoxications caused by these HMs.

Levels of HM in the atmosphere, in the occupational environment, and in biological samples can all be used to measure the impact of HM exposure on the health of workers and of the general populations residing in industrial areas [215]. Acute exposure to HMs can severely damage lungs, liver, kidneys, and the central nervous system (ATSDR, 2004). Long-term exposure can lead to HM bioaccumulation not only in humans, but also in crops, soil, and wildlife used as food sources, indirectly affecting humans [218,219]. While acute exposures to HMs knowingly causes severe toxic effects, concerns by public health authorities are currently focused on chronic, low-dose exposures, which can lead to cumulative effects. Even exposures to HMs at levels lower than those assigned for “safe” threshold values are potentially toxic, resulting in cancer, neurological damage, and infertility, among other effects [220,221,222].

Exposure to HMs can cause disturbances in the hematological system and can be used as biological end-points for assessing HM exposure; HM intoxications cause immunosuppression, anemias, and leukemias and disrupt coagulation [223,224,225,226]. While HM exposure can directly impact circulating blood cells, several toxic effects occur in the BM environment and affect hematopoiesis.

#### 2.5.1. Lead (Pb)

Exposure to Pb reduces the number of blood cells, including both erythrocytes and leukocytes. Toxic effects on BM due to Pb exposure have been described even at lower levels in humans under occupational exposures or in animal experimental intoxication models [227]. In this context, mice exposed to Pb have displayed reduced numbers of colony-forming units in their BM [228], and exposure to Pb impaired differentiation of CMPs, resulting in decreased numbers of mature myeloid cells [229]. Mechanisms associated with these effects involve higher expressions of interferon regulatory factor-8 (IRF8), which blocks C/EBPα and modulates neutrophil differentiation [230]. Recent evidence shows that exposure to Pb at occupational levels can result in lower numbers of innate lymphoid cells (ILCs) in the blood of mice, with this effect being dependent on activation of Janus Kinase-1 leading to an inability of BM CLP progenitors to become mature and be delivered into the bloodstream [231]. CLPs differentiate into innate lymphoid cell-restricted progenitors via transcription factor ID2, which further differentiate into mature ILC 1, 2, and 3 [232]. Under stimulation, mature ILC 1, 2, and 3 are activated and exert functions similar to those exerted by Th1, Th2, and Th17 cells, respectively [233,234]. These functions play roles during the innate immune response occurring in certain scenarios, such as during asthma, tumors, and tissue remodeling [235,236], all of which are aggravated due to Pb exposure.

Pb intoxication also interferes with erythropoiesis, as evidenced by aplastic anemia. High levels of reactive oxygen species seem to be the mediators of this effect, as children with aplastic anemia are reported to show higher levels of Pb in the blood alongside increased markers of oxidative stress [237].

#### 2.5.2. Cadmium (Cd)

Humans are exposed to Cd derived from occupational activities, cigarette smoking, Cd-contaminated dust and ingestion of contaminated food [238]. Cd is extensively distributed to other tissues and has a long half-life (approximately 10 years), contributing to its high toxicity [239]. Much like Pb exposure, Cd exposure affects the differentiation and functionality of HSCs in BM. It has been reported that mice exposed to Cd showed increased myelopoiesis to the detriment of lymphopoiesis, there being higher numbers of neutrophils and lower numbers of B and T lymphocytes in the bloodstream and in secondary lymphoid tissues. Cd exposure is also harmful to HSC niches in BM, as transplantation of normal HSCs to Cd-exposed and lethally irradiated mice did not repopulate the BM, whereas HSCs from Cd-exposed mice partially reconstituted the hematopoietic system of non-exposed and lethally irradiated mice [240]. A recent study carried out in mice further demonstrated that Cd exposure impaired the HSC ability to repopulate the BM of lethally irradiated recipients, and this toxic effect upon HSCs is dependent on increased expression of cdc42, a small GTPAse crucial for HSC functions in mice. Indeed, pharmacological blocking of cdc42 restored the hematopoietic ability of HSCs from Cd-exposed mice [241].

#### 2.5.3. Arsenic (As)

As is considered to be among the metals most capable of causing harmful effects to human health. Geologically distributed in pentavalent (As^5+^, arsenate) and trivalent (As^3+^, arsenite) forms in some regions of the world, its concentration in soil and water exceeds in up to 10 times allowed levels, according to the World Health Organization [242]. The most common form of contact with As is through contaminated water consumed either directly or through food. Absorption via inhalation also occurs during the handling of pesticides, fungicides and paints. Arsenic metabolism determines its toxicity, as trivalent arsenic, either methylated or not, easily reacts with thiol groups in proteins and is thus more toxic than pentavalent arsenicals [243].

Studies using murine models have found that exposure to arsenic in drinking water can result in anemia and impaired immune responses elicited by mononuclear cells [244,245,246,247,248]. These effects have also been confirmed in humans [249]. As can be easily distributed to several tissues through the bloodstream, reaching BM where it causes toxic effects. Indeed, arsenic trioxide (As_2_O_3_) administered to mice severely damages the BM microenvironment, making stromal cells unable to form a healthy matrix to support hematopoietic progenitors [250].

Epidemiological studies carried out in Bangladesh and Romania, areas characterized by geogenic contamination of underground drinking water, correlated chronic As exposure with anemia [251,252,253,254]. A case report demonstrated pronounced histological alterations in the BM of a patient suffering from arsenic poisoning, characterized by marked nuclear aberrations involving nucleus shape, chromatin distribution, and nuclear envelope [255]. In vitro analyses showed that the molecular mechanism for toxicity of arsenic trioxide in erythroleukemic cell lines and on normal hemopoietic progenitor cells (HPCs) involves several pathways, such as inhibition of Stat5 activation and reduced expression of target genes Bcl-X(L) and glycophorin-A; activation of apoptotic mechanisms leading to cleaving of erythroid transcription factors Tal-1 and GATA-1, whose integrity is required for erythroid cell survival and differentiation; and reduced expression of heat shock protein 70, which is required for maintaining GATA-1 integrity [256]. In vivo, exposure to arsenite also impaired the formation of burst-forming unit-erythroid (BFU-E) colonies and the differentiation of erythroblasts into further stages in mice [257].

#### 2.5.4. Mercury (Hg)

Mercury intoxications usually occur due to acute exposures to its natural form during extraction of fossil fuels, burning of biomass, forest fires, and deforestation [258]. However, exposure to small concentrations, which occurs during contact with dental amalgams, consumption of fish and other seafood from contaminated regions, and occupational exposure (e.g., farming, industrial activities, and gold mining), can also affect human health [259]. Hg is toxic to virtually every human organ; due to its affinity for sulfhydryl groups, Hg alters tertiary and quaternary structures of proteins and disrupts membrane permeability [259]. Disturbance of hematopoietic systems is also a hallmark of Hg intoxication, characterized mainly by anemia and lymphocytopenia [223,260].

The harmful effects of Hg on BM were first demonstrated in patients with BM hypoplasia [261]. Exposure of mice BM cells to inorganic and organic Hg inhibited colony formation [262], and exposure to Hg in rat BM cells inhibited activities of acetylcholinesterase, glutathione reductase, and glucose-6-phosphate [263,264]. Even though absorbed Hg reaches BM (Dabrowski et al., 1983), in vivo toxicity seems to be dependent on higher exposure doses and on frequent exposures, as low dose exposures caused only minor and transient impairments on lymphocyte production in mice [265,266]. Recently, toxic mechanisms associated with long lasting exposure to Hg in mice have been linked to a decreased proliferation of HSC, which is dependent on reduced levels of interferon gamma in BM [225].

Intoxications caused by HM, especially Pb, Cd, As, and Hg, have severe harmful effects on the hematopoietic systems, leading to anemia and immune deficiencies, and it is intriguing that mechanisms linked to such toxic effects have not yet been more thoroughly assessed. These metals reach BM and easily interact with proteins, affecting several hematopoietic pathways. Further studies on cellular and molecular mechanisms linked to the toxic actions of metals in BM should investigate additional toxic effects arising from such interactions.

## 3. Conclusions

**Figure 2:**
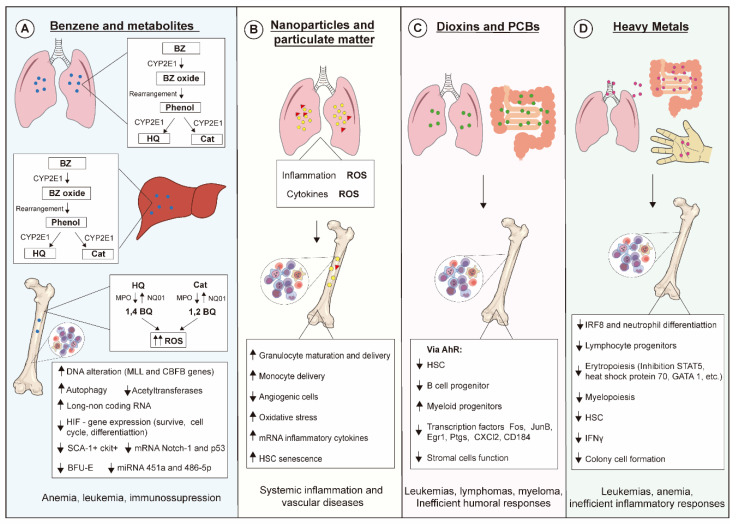
Molecular and cellular mechanisms linked to toxic effects of environmental pollutants on hematopoiesis. (**A**) Benzene (BZ) is metabolized by CYP2E1 in liver and lung generating hydroquinone (HQ) and catechol (Cat), which are then transformed into benzoquinone (BQ). These metabolites exert myelotoxic actions upon several hematopoietic progenitors mainly by increasing levels of reactive oxygen species (ROSs), leading to oxidative DNA damage. (**B**) Nanoparticles and particulate matter induce generation of ROSs and secretion of inflammatory cytokines that affect the behavior of several hematopoietic cell lineages. (**C**) Dioxins and PCBs bind to and activate the aryl hydrocarbon receptor (AhR) in mucosal tissues, modulating the hematopoietic stem cell (HSC) pool and triggering both immunosuppressive effects and myelodysplastic and malignant abnormalities. (**D**) Heavy metals can enter the body via several contact routes, their toxic effects being responsible for impairing inflammatory responses and triggering leukemias and anemias.

Exposure of living beings to environmental pollutants has increased, influencing public health policy-making even in scenarios where exposures are below thresholds considered safe. Simultaneous exposure to different air pollutants also certainly contributes to the increase of alarming data, which has been evidenced in epidemiological studies. Hematopoiesis is pivotal for hemostasis and host defense, and disturbances on this process lead to severe outcomes, as summarized in Figure 2. Advances in scientific knowledge regarding hematopoiesis mechanisms have evidenced novel targets for actions of xenobiotics not yet described, hence further studies on hematopoiesis are needed for improving environmental pollutant risk assessment.
